# The immature human ovary shows loss of abnormal follicles and increasing follicle developmental competence through childhood and adolescence

**DOI:** 10.1093/humrep/det388

**Published:** 2013-10-17

**Authors:** R.A. Anderson, M. McLaughlin, W.H.B. Wallace, D.F. Albertini, E.E. Telfer

**Affiliations:** 1 MRC Centre for Reproductive Health, Queens Medical Research Institute, University of Edinburgh, 47 Little France Crescent, Edinburgh EH16 4TJ, UK,; 2 Centre for Integrative Physiology, University of Edinburgh, Hugh Robson Building, EdinburghEH8 9XD, UK,; 3 Department of Paediatric Oncology, Royal Hospital for Sick Children, Edinburgh EH9 1LF, UK; 4 Institute for Reproductive Health and Regenerative Medicine, Kansas University Medical Centre, Kansas City, KS 66085, USA

**Keywords:** childhood, follicle, ovary, puberty, adolescence

## Abstract

**STUDY QUESTION:**

Do the ovarian follicles of children and adolescents differ in their morphology and *in vitro* growth potential from those of adults?

**SUMMARY ANSWER:**

Pre-pubertal ovaries contained a high proportion of morphologically abnormal non-growing follicles, and follicles showed reduced capacity for *in vitro* growth.

**WHAT IS KNOWN ALREADY:**

The pre-pubertal ovary is known to contain follicles at the early growing stages. How this changes over childhood and through puberty is unknown, and there are no previous data on the *in vitro* growth potential of follicles from pre-pubertal and pubertal girls.

**STUDY DESIGN, SIZE, DURATION:**

Ovarian biopsies from five pre-pubertal and seven pubertal girls and 19 adult women were analysed histologically, cultured *in vitro* for 6 days, with growing follicles then isolated and cultured for a further 6 days.

**PARTICIPANTS/MATERIALS, SETTING, METHODS:**

Ovarian biopsies were obtained from girls undergoing ovarian tissue cryopreservation for fertility preservation, and compared with biopsies from adult women. Follicle stage and morphology were classified. After 6 days in culture, follicle growth initiation was assessed. The growth of isolated secondary follicles was assessed over a further 6 days, including analysis of oocyte growth.

**MAIN RESULTS AND THE ROLE OF CHANCE:**

Pre-pubertal ovaries contained a high proportion of abnormal non-growing follicles (19.4 versus 4.85% in pubertal ovaries; 4004 follicles analysed; *P* = 0.02) characterized by indistinct germinal vesicle membrane and absent nucleolus. Follicles with this abnormal morphology were not seen in the adult ovary. During 6 days culture, follicle growth initiation was observed at all ages; in pre-pubertal samples there was very little development to secondary stages, while pubertal samples showed similar growth activation to that seen in adult tissue (pubertal group: *P* = 0.02 versus pre-pubertal, ns versus adult). Isolated secondary follicles were cultured for a further 6 days. Those from pre-pubertal ovary showed limited growth (*P* < 0.05 versus both pubertal and adult follicles) and no change in oocyte diameter over that period. Follicles from pubertal ovaries showed increased growth; this was still reduced compared with follicles from adult women (*P* < 0.05) but oocyte growth was proportionate to follicle size.

**LIMITATIONS, REASONS FOR CAUTION:**

These data derive from only a small number of ovarian biopsies, although large numbers of follicles were analysed. It is unclear whether the differences between groups are related to puberty, or just age.

**WIDER IMPLICATIONS OF THE FINDINGS:**

These findings show that follicles from girls of all ages can be induced to grow *in vitro*, which has important implications for some patients who are at high risk of malignant contamination of their ovarian tissue. The reduced growth of isolated follicles indicates that there are true intrafollicular differences in addition to potential differences in their local environment, and that there are maturational processes occurring in the ovary through childhood and adolescence, which involve the loss of abnormal follicles, and increasing follicle developmental competence.

**Study funding/competing interest(s):**

Funded by MRC grants G0901839 and G1100357. No competing interests.

## Introduction

Human primordial follicle formation is completed in fetal life (with primordial follicles present from 17 weeks of gestation) and is rapidly followed by the initiation of follicle growth, such that all stages of pre-antral follicles and antral follicles other than pre-ovulatory stages have been reported before puberty (Lintern-Moore *et al*., 1974; Peters *et al*., 1978). The rise in gonadotrophin secretion during puberty results in the progression of all stages of follicle growth and the onset of ovulation, but there are few data relating to developmental changes in the ovary between follicle formation and post-pubertal, adult function. This reflects the limited availability of samples for histological analysis, and the absence until recently of any serum marker of early follicle growth. The advent of measurement of anti-Müllerian hormone (AMH) has allowed the demonstration that there is a neonatal rise in follicular activity comparable with the well-established male ‘mini-puberty’ of the neonate (Kuiri-Hanninen *et al*., 2011). The currently available histological data indicate a steady rise in follicular growth through childhood without a dramatic change at the onset of puberty (Peters *et al*., 1976). This is reflected in rising AMH concentrations through childhood, but there is a transient plateau or decline in AMH in adolescence (Kelsey *et al*., 2011; Hagen *et al*., 2012) suggesting non-linear changes in the rate and extent of follicle development. The changes in ovarian activity underlying these observations are unknown, and require a combination of anatomical and functional studies to address. Thus, while the pattern of follicle growth in pre-pubertal ovaries has been reported to be similar to adults (with the exception of absent pre-ovulatory follicles) (Lintern-Moore *et al*., 1974; Peters *et al*., 1976; Peters *et al*., 1978), exceptions to this have been attributed to underlying systemic illness and anticancer treatments (Himelstein-Braw *et al*., 1977; Himelstein-Braw *et al*., 1978) rather than fundamental maturational processes that normally occur only shortly before the onset of adult ovulatory function. Developmentally competent oocytes have been obtained from antral follicles in young girls (Revel *et al*., 2009) but no previous studies have explored directly the *in vitro* growth potential of follicles from children and adolescents.

Ovarian tissue cryopreservation is emerging as a potential method for fertility preservation for adult women. At least 23 live births have been reported following reimplantation of frozen/thawed ovarian tissue, some following natural conception and others involving assisted reproduction (Donnez *et al*., 2013). In girls and adolescents, for whom ovarian stimulation for oocyte/embryo cryopreservation is inappropriate, ovarian tissue cryopreservation is currently the only option for fertility preservation and small series of patients in whom this has been performed have been reported (Poirot *et al*., 2002; Anderson *et al*., 2008; Jadoul *et al*., 2010). There are now two case reports of hormonal activity following ovarian tissue replacement in adolescents to induce puberty (Poirot *et al*., 2012; Ernst *et al*., 2013), although this indication is controversial (Anderson *et al*., 2013). While this demonstrates the potential for hormonal activity reflecting follicle growth, the potential for fertility restoration, the key goal of this invasive and experimental treatment, is unknown.

In some cases the ovarian tissue removed for fertility preservation carries a risk of (or is actually demonstrated to have) contamination with cancer cells (Abir *et al*., 2010; Dolmans *et al*., 2010; Rosendahl *et al*., 2010). Therefore, *in vitro* follicle growth strategies are required to utilize the population of oocytes contained in this tissue. Activation of adult non-growing human ovarian follicles to the antral stage of development has been achieved (Telfer *et al*., 2008). If this can be combined with systems already established to complete the final stages of oocyte growth and maturation *in vitro*, fertility restoration is a realistic approach possibly for patients for whom ovarian tissue reimplantation is potentially hazardous (Telfer and Zelinski, 2013). Whether these techniques can be applied directly to ovarian tissue from children and adolescents has not been investigated.

The purpose of this study was to investigate human folliculogenesis by reassessing *in vivo* ovarian maturation and follicle development in childhood and adolescence and to determine whether follicles from girls activate, survive and develop *in vitro* in a two-step culture system in a manner comparable with follicles from adult women.

## Materials and Methods

### Ovarian cortical tissue

Biopsies were obtained laparoscopically from 12 young patients undergoing removal of ovarian cortex for fertility cryopreservation prior to chemotherapy or radiotherapy for malignant disease or chronic illness (Table I). Protocols for both fertility preservation and donation for research had Ethical Committee approval, and all patients and/or their parents gave informed consent to both aspects in writing. The mean patient age was 11.4 ± 1.0 years (mean ± SEM) with a range of 3.0–16.0 years. For most analyses patients were divided into two groups: those showing no signs of puberty (aged 3.0–12.2 years, *n* = 5) and those in early or established puberty (12.0–16.0 years old, *n* = 7; hereafter termed ‘pubertal’). Samples were treated identically to adult human ovarian biopsies (Telfer *et al*., 2008) and data are compared with results obtained from contemporaneous ovarian biopsies (*n* = 19) obtained from adult women undergoing Caesarean section (age range 25–38 years).

**Table I:** Diagnoses and ages of patients from whom tissue was obtained

Diagnosis	Patient age (years)	Biopsy condition
Rhabdomyosarcoma	3.0	Fresh
Ependymoma	8.2	Fresh
Rhabdomyosarcoma	7.9	Cryopreserved
Rhabdomyosarcoma	10.6	Fresh
Ewing's sarcoma	12.2	Fresh
Ewing's sarcoma	12.0	Fresh
Wilm's tumour	12.3	Fresh
Sickle cell anaemia	14.6	Fresh
Acute myeloid leukaemia	14.4	Fresh
Hodgkin's lymphoma	14.1	Fresh
Hodgkin's lymphoma	15.3	Fresh
Rhabdomyosarcoma	16.0	Cryopreserved

All biopsies were received directly from surgery except for two patients whose tissue had been cryopreserved.

### Tissue preparation and fragment culture

Fresh ovarian cortical biopsies (∼8 × 5 mm, with variable thickness) were transported to the laboratory and prepared for culture as previously described (Telfer *et al*., 2008) with slight modification. Two of the biopsies had been cryopreserved by slow freezing (Gosden *et al*., 1994) for fertility preservation reasons and were donated for research post-mortem. Briefly, the tissue was transferred into fresh pre-warmed Leibovitz medium (GIBCO BRL, Life Technologies Ltd., Paisley, Renfrewshire, UK) supplemented with sodium pyruvate (2 mM), glutamine (2 mM), human serum albumin (HSA) (3 mg/ml), penicillin G (75 mg/ml) and streptomycin (50 mg/ml); all chemicals were from Sigma Chemicals (Poole, Dorset, UK). The tissue was examined under light microscopy and any visible follicles were removed as well as any haemorrhagic or damaged areas. With the cortex uppermost, the tissue was then gently stretched using the blunt edge of a scalpel blade and any excess stromal tissue underneath excised. Finally, using an angled incision, the tissue was cut with a scalpel into fine tissue fragments ∼3 × 1× 0.5 mm, the largest dimension being the cortical surface. At least two fragments of tissue from each biopsy were immediately fixed in 10% neutral buffered formalin (NBF) for histological evaluation. Due to the highly variable distribution of follicles in the human cortex, fragments fixed as 0 h controls were twice as large as fragments which were cultured. Tissue fragments were cultured individually in 24-well cell culture plates (Corning B.V. Life Sciences Europe, Amsterdam, The Netherlands) containing serum-free McCoy's 5A medium with bicarbonate supplemented with HEPES (20 mM; Invitrogen Ltd, Paisley, UK), glutamine (3 mM; Invitrogen), HSA (0.1%), penicillin G (0.1 mg/ml), streptomycin (0.1 mg/ml), transferrin (2.5 µg/ml), selenium (4 ng/ml), human insulin (10 ng/ml), recombinant human FSH (1 ng/ml) and ascorbic acid (50 µg/ml); all from Sigma Chemicals unless otherwise specified. Fragments were cultured for 6 days at 37 °C in humidified air with 5% CO_2_, with media changed every 2 days.

On completion of the culture period fragments were observed under light microscopy and those containing growing follicles with a diameter of ∼100 µm were transferred to pre-warmed Leibovitz medium, as described above, for mechanical dissection of follicles. The remaining tissue fragments were fixed in NBF for histological and confocal analyses.

### Thawing of cryopreserved cortical tissue

Slow frozen tissue was thawed as described (Gosden *et al*., 1994) with slight modification. Briefly, cryovials were removed from liquid nitrogen and exposed to the air for 1 min before being plunged into a 37°C water bath for 2 min. The cryoprotectant was then removed and replaced with pre-warmed (37°C) Leibovitz medium supplemented as described above but without HSA and with 10% human serum (Bioreclamation, Inc., Hicksville, New York) and 1.5 M dimethyl sulphoxide (DMSO) (Sigma Chemicals) (Solution 1). Cryovials were gently agitated in this solution for 5 min. Solution 1 was then removed and replaced with Solution 2 (Leibovitz with supplements as per Solution 1 but with a reduced concentration of 1 M DMSO), and gently agitated for 5 min. Solution 2 was removed and replaced with Solution 3 (Leibovitz with supplements as per Solution 2 with HSA (3 mg/ml) and a lower DMSO concentration of 0.5 M, but without human serum). After a further 5 min agitation Solution 3 was removed and replaced with Leibovitz medium supplemented as described above for dissection. Cryovials were gently agitated in this solution for 5 min, thereafter the tissue was transferred into a Petri dish containing fresh dissection medium and prepared for culture as described above for fresh tissue.

### Isolation and culture of pre-antral follicles

After 6 days in culture, pre-antral follicles were visible under the dissecting microscope, and were dissected from cortical fragments using 25G needles as previously described (Telfer *et al*., 2008). A total of 88 follicles ranging in diameter from 82 to 140 µm (mean 107 ± 2.3 µm) were isolated. The number of follicles dissected varied from fragment to fragment and biopsy to biopsy. Isolated follicles were placed individually in 96-well, V-bottomed culture plates (Corning Costar Europe, Badhoevedorp, The Netherlands) in 150 µl of culture media as described earlier for cortical fragment culture, supplemented with 100 ng/ml activin A (R & D Systems, Abingdon, UK). Isolated follicles were incubated for 6 days at 37°C in humidified air with 5% CO_2_. Half of the culture medium was removed and replaced with fresh every second day and follicle diameter recorded.

### Histological analysis

Cortical fragments and isolated follicles were fixed in NBF for 48 h. Fixed tissue was then dehydrated through increasing concentrations of alcohol (70, 90 and 100%) and immersed in cedar wood oil for 24 h. Cedar wood oil was cleared from the tissue using toluene for 30 min and the tissue fragments/follicles were then individually immersed in paraffin wax at 60°C for 4 h with wax changes every hour to ensure toluene clearance. Tissue was sectioned at 6 µm, mounted on slides and allowed to dry overnight prior to staining with eosin and haematoxylin.

### Assessment of cortical fragments

Every section of every cortical fragment was examined under light microscopy. Follicles were categorized according to their developmental stage and their morphological normality, and their number determined in relation to the volume of ovary examined as described (Lass *et al*., 1997). Briefly, tissue volume was calculated using the formula:
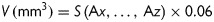

where *S*(A*x*, … A*z*) is the sum of the area of all tissue sections analysed per patient and 0.06 is the distance between sections. Follicle density was determined by dividing the total number of follicles per patient by the tissue volume and expressing this value as follicles per mm^3^. Follicle maturity was based on granulosa cell configuration (Telfer *et al*., 2008). Follicle morphology was considered normal if the oocyte was generally spherical, the resting prophase germinal vesicle was located centrally and contained a visible nucleolus, the oolemma was intact, the ooplasm was evenly distributed, <10% of associated granulosa cells were pyknotic and the basal lamina was intact. Only sections of follicles containing the nucleolus were assessed to avoid double counting.

### Assessment of isolated follicles

Histological sections of follicles containing the nucleolus were assessed using a light microscope with a crossed micrometer scale. Follicle and oocyte diameters were measured as well as the percentage of pyknotic granulosa cells, and an assessment was made of oocyte integrity as described above.

### Immunofluorescence and confocal microscopic analysis

Cortical ovarian pieces measuring 1 × 1 × 0.5 mm were fixed for 24 h at 4°C in microtubule stabilization buffer extraction fix (MTSB-XF) or 4% paraformaldehyde and samples were stored in blocking buffer (Rodrigues *et al*., 2009) at 4°C for 3–10 weeks prior to labelling. Because of the thickness of cortical ovarian pieces, extended periods of extraction, labelling and washing were required to facilitate reagent penetration and ensure adequate washout of excess immunological probes. This was achieved by an initial 24 h wash at 4°C in 1 ml of a blocking solution containing 0.5% Triton-X-100 on a shaking platform and the same solution was used for all subsequent labelling and wash steps. Primary antibody labelling with a 1:100 dilution of mouse monoclonal anti-acetylated alpha tubulin (InVitrogen) was performed for 24 h in a 100 µl volume at 4°C followed by three 4 h washes in a blocking buffer volume of 1 ml. The same conditions were used for labelling with goat anti-mouse immunoglobulin G conjugated with AlexaFluor 488 containing 1 µg/ml Hoechst 33258 followed by three wash steps over a 18 h time period that included the Hoechst dye. A penultimate wash with 10 units/ml AlexaFluor 568 phalloidin was used to label f-actin and samples were mounted in a glycerol-based medium containing Hoechst dye and 2% sodium azide as an anti-fade reagent.

For confocal imaging, Z-stack data sets were collected with ×20 or ×40 objectives at 0.5–1.0 µm steps using excitation lines of 405, 488 or 568 nm for the detection of Hoechst 33258, AlexaFluor 488-tubulin or AlexaFluor 568-phalloidin, respectively, using a Zeiss LSM 510 instrument.

### Statistical analyses

Mean follicle and oocyte diameter measurements were compared using one-way analysis of variance with *post hoc t*-tests. The proportions of the developmental stages observed pre- and post-culture were compared using *χ*^2^ analysis and the numbers of follicles at different stages were compared using the Mann–Whitney *U*-test. Normal follicle morphology was expressed as a proportion of those follicles present at each corresponding stage of development before and after culture, and groups were compared using the *χ*^2^ test.

## Results

### Follicle number and morphology in cortical fragments

**Figure 1:**
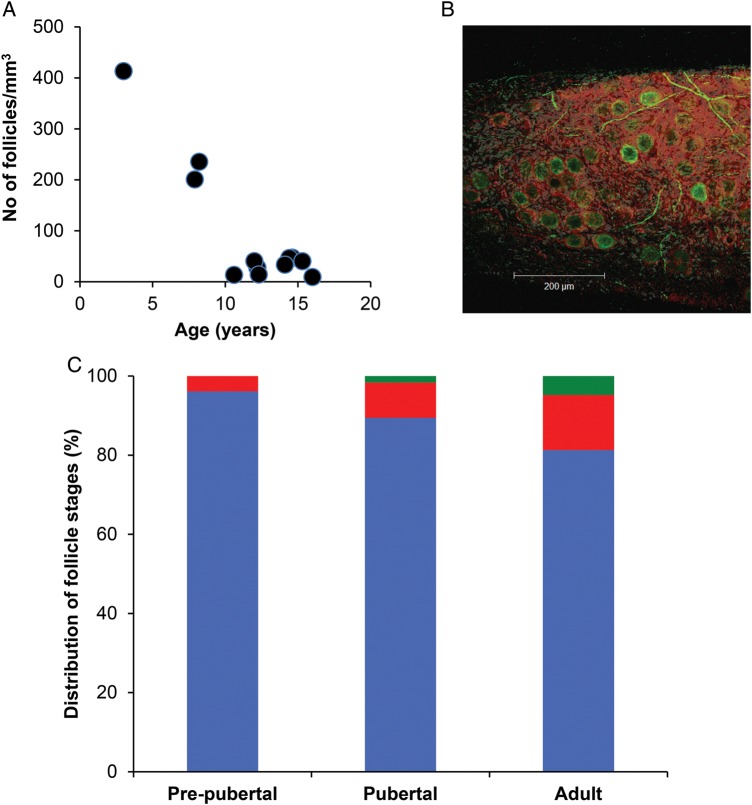
(**A**) Relationship between follicle number (per mm^3^ of tissue) and age. (**B**) Confocal image of ovarian cortex from a 3-year-old girl immunostained for acetylated tubulin (green) and f-actin (using phalloidin: red). (**C**) Distribution of follicle classes (as percentage of total) in ovarian tissue from pre-pubertal and pubertal girls, and adults. Blue: non-growing follicles; red: primary follicles; green: secondary follicles. A total of 2672, 1278 and 798 follicles are classified in the three age groups, respectively.

To determine the number and developmental stage of follicles in ovarian cortical tissue from children and teenagers, 31 fragments of tissue (2–3 per patient) were fixed fresh or post-thawing and processed for histological examination. A total of 4004 follicles were analysed in uncultured ovarian tissue. Follicle number was much higher in the three youngest girls, at 201–413/mm^3^, whereas it ranged from 9 to 46/mm^3^ in the remainder (Fig. 1A). Using whole mount confocal microscopy, it was apparent that the great majority of follicles in uncultured tissue were confined to a region subtending the tunica albuginea, recognizable because of the lower cell density and absence of nerve fibres (Fig. 1B). Most follicles appeared to be of a uniform diameter and non-growing, and embedded in an actin-rich dense stroma. Notably, the acetylated tubulin labelling not only rendered the nerve fibre network but also labelled the follicles themselves. Additionally, the pattern of phalloidin staining showed a dense layer of f-actin at the outer limits of follicles. While a small number of secondary follicles were observed in tissue from the pubertal group, none were found in the pre-pubertal group (Fig. 1C).

**Figure 2:**
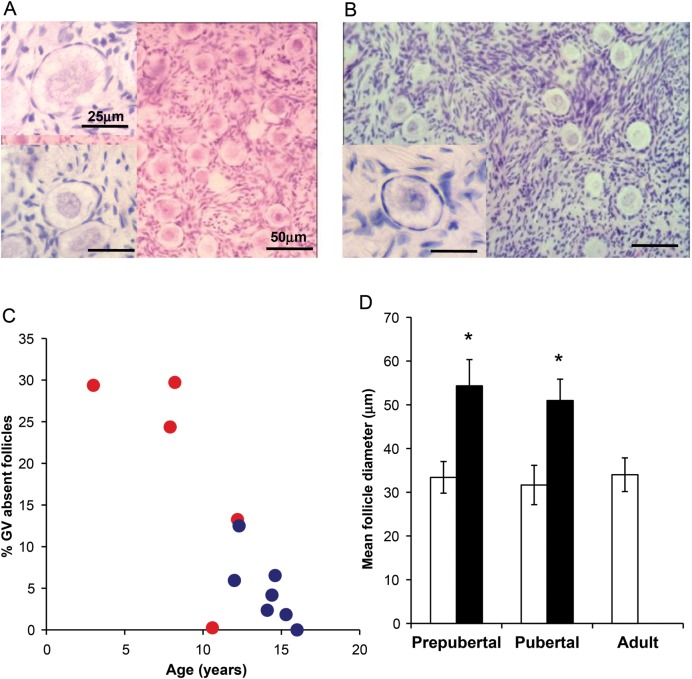
(**A**) Photomicrograph of ovarian tissue from pre-pubertal girl aged 8 years. Non-growing follicles with both abnormal (top inset) and normal (bottom inset) morphology are present. (**B**) Photomicrograph of ovarian tissue from pubertal girl aged 14 years showing only follicles of normal morphology (main image and inset). Scale bars 25 µm in inserts, 50 µm in main images. (**C**) Relationship between percentage of non-growing follicles with abnormal morphology and age. Red circles: pre-pubertal girls, blue circles, pubertal girls. (**D**) Diameter of normal (white bars) and abnormal (black bars) non-growing follicles in pre-pubertal and pubertal groups, and in adult ovary (which contains no abnormal non-growing follicles). Mean ± SEM, pre-pubertal group normal *n* = 2079, abnormal morphology *n* = 500; pubertal group normal *n* = 1357, abnormal morphology *n* = 68; adult *n* = 44, *P < 0.05 versus normal.

Analysis of follicle health showed that in biopsies from the pre-pubertal group a significant number of the oocytes in non-growing follicles showing an abnormal morphology with absent nucleolus and poor germinal vesicle definition (Fig. 2A; normal morphology represented in lower inset and in Fig. 2B). There was a relationship between the prevalence of these abnormal follicles and age (Fig. 2C); they constituted 19.4 ± 5.6% of oocytes within non-growing follicles in tissue from the pre-pubertal group and 4.8 ± 1.6% of non-growing follicles from the pubertal group (*P* = 0.02; Fig. 2C). One of the pre-pubertal girls (age 10.6 year) had a low total follicle number in relation to the rest of that group, and very few abnormal follicles. Follicles with absent nucleoli were significantly larger than morphologically normal non-growing follicles (54.3 ± 6.0 versus 33.4 ± 3.6 µm in pre-pubertal, *n* = 2579 and 51.0 ± 4.9 versus 31.7 ± 4.5 µm in pubertal ovaries *n* = 1425; both *P* < 0.05) (Fig. 2D). These abnormal follicles were never observed in tissue from adult women (Fig. 2D); non-growing follicles of normal morphology were of the same size in the pre-pubertal and pubertal groups and in adult women.

### Follicle development in cultured cortical fragments

**Figure 3:**
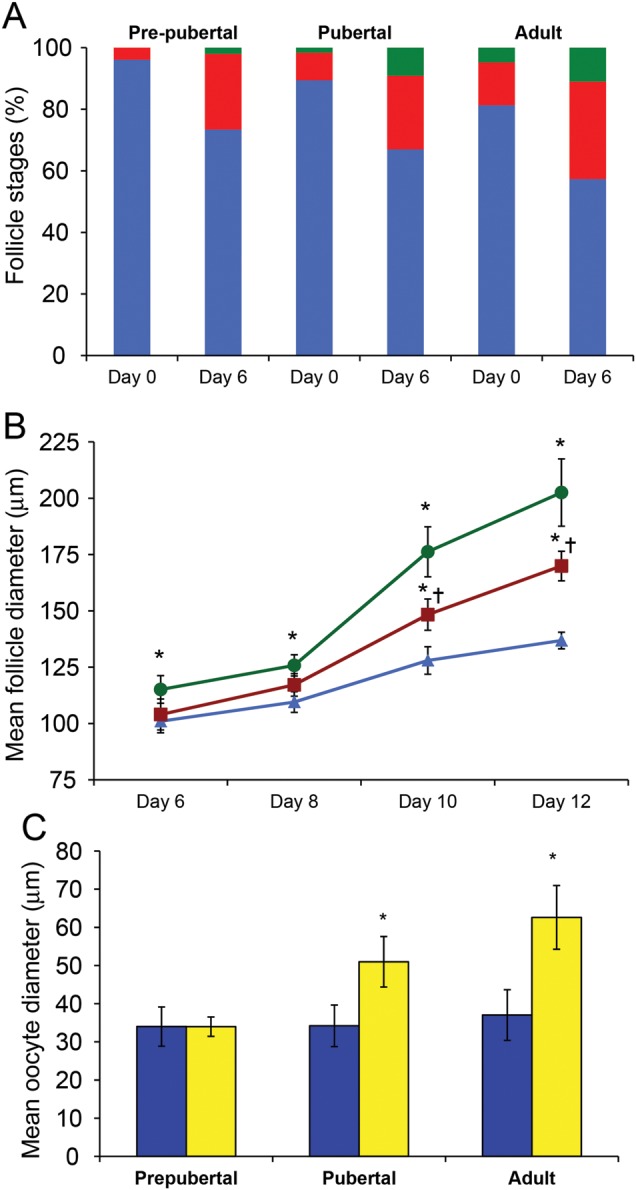
(**A**) Distribution of follicle stages (as percentage of total) in cultured ovarian tissue in pre-pubertal, pubertal and adult groups, before culture (Day 0) and after 6 days. Blue: non-growing follicles; red: primary follicles; green: secondary follicles. (**B**) Follicle diameter during culture of isolated secondary follicles. Follicles were isolated from tissue after 6 days of culture, and follicle diameter was determined 2, 4 and 6 days thereafter. Blue triangles, pre-pubertal group; red squares, pubertal group; green circles: adult group (mean ± sem, *n* = 15, 73 and 44 respectively). *P < 0.05 versus pre-pubertal group, ^†^*P* < 0.05 versus adult group. (**C**) Oocyte diameter of isolated secondary follicles after culture for 6 days in tissue, then a further 6 days as isolated follicles, to Day 12. Blue columns: Day 6; yellow columns, Day 12. Mean ± SEM, *n* = 15 and 77 follicles in pre-pubertal and pubertal groups, respectively, *n* = 44 follicles in adult group.*P < 0.05 versus Day 6.

Non-growing follicles accounted for a mean of 96 and 89% of all the follicles observed in uncultured tissue in pre-pubertal and pubertal groups, respectively (2672 and 1278 follicles examined in each of the groups); these were not significantly different, and was also similar to the proportion in adult ovary (81%, *n* = 798 follicles examined) (Fig. 1C). After 6 days of incubation, cortical fragments were examined microscopically and the stage of follicle development categorized. A total of 1500 and 1467 follicles were analysed in the pre-pubertal and pubertal groups, respectively, and 592 in the adult group. Initiation of follicle growth was observed in all cultured biopsies. After 6 days of incubation the proportion of non-growing follicles was 73.4% in the pre-pubertal group and 66.9% in the pubertal group (both *P* < 0.001 versus uncultured) and 57.3% in the adult group (*P* < 0.001 versus uncultured; Fig. 3A). Development to the secondary stage occurred more frequently in the pubertal group with 9.1% of follicles reaching this stage at Day 6 culture (from 1.6% in uncultured tissue; *P* = 0.007), whereas only 2.0% of follicles were at that stage in the pre-pubertal group compared with 0% in uncultured (not significant versus uncultured; *P* = 0.02 versus pubertal group after 6 days; Fig. 3A). In adult tissues, 11.0% of follicles had reached the secondary stage after culture compared with 4.8% at the start (*P* = 0.03; Fig. 3A). Thus, pre-pubertal and pubertal samples were similar pre-culture, and while the pre-pubertal samples showed limited follicle growth activation in culture, the pubertal samples showed similar activation to that seen in adult tissue.

### Growth and survival of isolated follicles

After 6 days of culture 73 growing follicles were isolated from the pubertal group with follicles being isolated from every biopsy. In contrast only 15 follicles were obtained from the pre-pubertal group and all of them were from one 8-year-old girl. These were compared with 44 follicles isolated from the adult group. The size of freshly isolated follicles after initial tissue culture was similar for all specimens (100–110 µm), although those from the pre-pubertal girl were significantly smaller than those from adult women (101 ± 5 versus 115 ± 6 µm, *P* < 0.05). A significant increase in mean follicle diameter occurred during the subsequent 6-day culture period in all groups (Fig. 3B) but the rate of growth differed between groups. Whilst isolated follicles from the pre-pubertal girl increased in diameter (to 137 ± 4 µm) this was less than follicles isolated from the pubertal group (104 ± 7 to 170 ± 7 µm; *P* < 0.05 versus pre-pubertal, Fig. 3B). Furthermore, follicles isolated from the pubertal group did not grow at the same rate as follicles isolated from adult ovarian tissue, which reached 203 ± 15 µm (*P* < 0.05 versus both groups, Fig. 3B).

**Figure 4:**
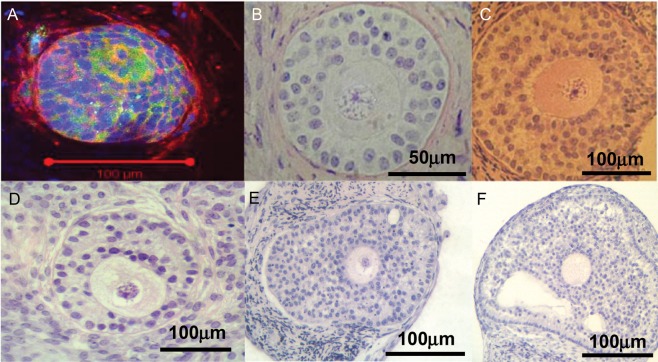
Photomicrographs of secondary follicles after 6 days of isolated follicle culture. (**A**) immunofluorescent image of an abnormal follicle from a pre-pubertal girl (age 8 years); acetylated tubulin (green) and f-actin (red) with DAPI nuclear counterstain (blue). (**B**, **C** and **D**) are secondary follicles from pubertal girls (ages 12.0 and 15.3 years) showing healthy morphology after culture; (**E**) and (**F**) are follicles from adult ovary (ages 31 and 34 years), showing early antrum formation. Note that in all cases some ovarian stroma remains attached to the individually dissected follicles.

Oocyte diameter at the end of the 6 days of isolated follicle culture was measured in each follicle. This revealed that whilst an increase in follicle diameter had been observed in follicles isolated from the pre-pubertal girl, no significant oocyte growth had occurred during this period (Fig. 3C). Significant oocyte growth (as well as the above-described follicle growth) was observed in follicles isolated from the pubertal group (*P* < 0.05) (Fig. 3C). While this was less than that observed in follicles from adult women (*P* < 0.05) (Fig. 3C) it may be appropriate for the degree of follicle growth achieved as in both pubertal and adult follicles oocyte diameter was ∼30% of follicle diameter at the end of culture. Multilaminar follicles were observed after the culture period in follicles from both the pre-pubertal and pubertal groups (Fig. 4A, B, C), but none formed antral cavities under these conditions. This contrasts with adult follicles of which ∼30% develop antral cavities under these culture conditions, at a follicle diameter of ≥200 µm (Telfer *et al*., 2008). This corresponds to the size range of 200–400 µm at which antral cavities develop *in vivo* (Gosden and Telfer, 1987). As the mean diameter of follicles at the end of the culture period was 137 ± 4 µm and 170 ± 7 µm in the pre-pubertal and pubertal groups, respectively, the absence of antral cavities appears to reflect the limited growth achieved over the time period studied.

Some of the follicles isolated from the youngest tissue showed the abnormal oocyte morphology described above. These follicles showed an asymmetric disposition of somatic cells compared with the more centrosymmetric disposition of the oocyte seen in normal follicles. A representative example of such a follicle is shown in Fig. 4A.

## Discussion

Very little is known about the pre-pubertal human ovary. Primordial follicles form during fetal life (Baker 1963; Fulton *et al*., 2005), and follicle growth is then initiated (Lintern-Moore *et al*., 1974; Peters *et al*., 1978). There is a transient increase in follicle growth and hormone production in the neonatal girl, followed by a period of relative quiescence until the rising gonadotrophins associated with puberty support later stages of follicle growth (Peters *et al*., 1976) and ovulatory cycles are ultimately established. The recent ability to monitor early follicle activity through the measurement of serum AMH has shown that there is a steady increasing follicle growth in the pre-pubertal years, with a poorly understood plateau after the onset of puberty (Kelsey *et al*., 2011; Hagen *et al*., 2012): these findings indicate that the post-natal ovary may not be merely a gonadotrophin-deficient version of the adult organ, but show intrinsic maturational changes in childhood. How these changes in ovarian composition and function impact on the long-term survival and developmental potential of the ovarian follicle reserve remains an unexplored area. Recent data have shown that young adult women have a higher prevalence of normal length but anovulatory cycles than older women (Hambridge *et al*., 2013), further suggesting that optimal follicle function is not achieved or acquired until at least the mid-20s. The need for greater understanding is highlighted by the developing practice for offering ovarian cryopreservation as a fertility preservation option to young girls to increase their chances for restoration of fertility (Poirot *et al*., 2002; Anderson *et al*., 2008; Jadoul *et al*., 2010).

This study demonstrates for the first time that there are striking differences in the follicle population that change with age and pubertal maturation. The observation of a significant population of what appear to be abnormal oocytes within primordial follicles in the younger girls has not been reported before and this may have implications for the use of such tissue. This population either degenerates or is utilized preferentially, as fewer are seen in pubertal girls and none were observed in any adult tissue. Furthermore, whilst primordial follicles in ovarian cortical tissue can be activated to grow *in vitro* at all ages, the initiation of follicle growth was low in the pre-pubertal group, and the rate of follicle growth was very limited in pre-pubertal girls, and suboptimal in the pubertal group, some of whom had exhibited regular menstrual cycles for up to 5 years before biopsy. Strikingly, while follicles grew slowly in both groups, oocyte growth appeared even more compromised, with no growth in the follicles from pre-pubertal ovary.

The presence of a large population of abnormal primordial follicles was clearly identified. These follicles were characterized by an indistinct germinal vesicle membrane and absent nucleolus, and were larger than those with normal morphology. Those follicles with normal morphology were of the same size as in adult tissue. These observations indicate that these abnormal follicles are a distinct population, rather than a variation on the normal. That their prevalence declines with age to being absent in adult ovary indicates that they are preferentially lost, perhaps through unknown ‘quality control’ mechanisms. The recent identification that two populations of primordial follicles are formed in the mouse ovary, an initial population with a more medullary location that is lost before sexual maturation and a second population, formed later and responsible for fertility (Mork *et al*., 2012), offers an intriguing parallel. Primordial follicle structure in the ovary of pre-pubertal girls was described by Baker (1963). He described atresia in diplotene oocytes in specimens from 4 girls aged 6 months to 7 years, with atretic oocytes almost as prevalent as normal ones (i.e. constituting 40–48% of the total oocyte pool) but with little difference in the prevalence between specimens and thus no clear evidence for a decline with age across the limited range examined. The atretic oocytes he described were approximately twice the diameter of normal ones and, at least at the earlier stages, retained a nuclear membrane thus do not correspond closely with the morphological features described here. A more recent study also described the primordial follicle population in ovaries of girls aged 4–16 years (Albamonte *et al*., 2013). No evidence of apoptosis in primordial follicles was found at any age examined, but a detailed histological analysis was not performed.

The potential of adult human ovarian follicles to develop from primordial stages to antral stages in an *in vitro* system has already been established (Telfer *et al*., 2008); however, to the best of our knowledge this is the first time childhood and adolescent follicle development *in vitro* has been reported. We categorized subjects according to whether they had entered puberty, based on clinical evaluation. While this is of physiological and clinical relevance, the distinction is used for convenience and does not indicate causality, as there were too few subjects in the study to make robust comments about any relationship between our findings and pubertal status. While these data demonstrate proof of principal that follicle growth can be supported *in vitro* even at very young ages, key differences from adult ovarian tissue have been demonstrated. First, while the proportion of follicles at the non-growing stage was very similar in the two groups, the rate of follicle growth initiation was low in the pre-pubertal group. Understanding of growth initiation is limited, although the biochemical pathways involved are increasingly being elucidated with the pten/PI3kinase pathway within the oocyte now recognized to be of central importance (Reddy *et al*., 2010). Additionally, it appears that primordial follicles inhibit the growth initiation of nearby follicles (Da Silva-Buttkus *et al*., 2009). As follicle density was much higher in the pre-pubertal biopsies, it is possible that resulting higher concentrations of one of more postulated inhibitory factors would reduce activation in those specimens.

Secondly, while oocyte and follicle growth of isolated follicles was observed in all samples across all ages, growth of both was significantly less than that from adult tissue. Thus, these follicles appeared to have an intrinsic growth defect. Oocyte growth is tightly coupled to follicle growth (Albertini and Barrett, 2003), thus the absence of oocyte growth in follicles from a pre-pubertal girl is a further indication that these isolated secondary follicles are different from those from adults. It was possible to isolate secondary follicles from only one girl in the pre-pubertal group, thus this result needs to be interpreted cautiously, but the inability to isolate such follicles from other girls in this group (compared with their ready isolation from the pubertal group) provides further evidence of the limited follicle development achieved in those specimens. Mean oocyte diameter achieved in the pubertal group after incubation was also lower than in isolated adult follicles activated from the non-growing state, but appeared proportional to follicle growth (Telfer and Gosden, 1987). This suggests that even after the onset of puberty and regular menstrual cycles, full follicle growth potential has not been achieved, although there appeared to be appropriate follicle/oocyte coordination. These data collectively indicate that there are very important maturational processes occurring in the ovary in the transition from childhood through puberty to adulthood which result in the loss of large numbers of abnormal follicles and the acquisition of the ability of the remaining, normal follicles to grow in an ‘adult’ manner. While the culture method used here supports adult follicle growth to antral stages, it is possible that modifications, for example to the growth factor components, may allow improved growth of follicles from pre-pubertal and pubertal ovary. Two case reports show the potential for follicle development (reflected in hormone production sufficient to induce puberty and menses) in pre-pubertal ovarian tissue after autografting (Poirot *et al*., 2012; Ernst *et al*., 2013). There is therefore clearly the potential of follicles from this age group to mature *in vivo*, although detailed analysis of early follicle activation and growth cannot be determined following transplantation.

The presence of growing follicles has been demonstrated previously in the ovaries of healthy infants and children as well as those diagnosed with malignancy (Lintern-Moore *et al*., 1974; Peters *et al*., 1975; Peters *et al*., 1976; Himelstein-Braw *et al*., 1977; Himelstein-Braw *et al*., 1978). This was ascertained by analysing sections of whole paediatric ovaries obtained at autopsy. In the present study a small piece of biopsied tissue was examined per patient. Follicle development appeared to be age dependent with no secondary follicles observed in freshly fixed or thawed fixed biopsies from pre-pubertal girls. Even in the pubertal group <1% of the follicles present were at the secondary stage of development. Although the ovarian cortex in childhood is richly endowed with follicles, their distribution within the tissue is recognized to be very variable (Qu *et al*., 2000; Schmidt *et al*., 2003) therefore the paucity of growing follicles seen in fixed uncultured tissue may be attributable to the quantity of tissue available for examination per patient. Tissue examined in this study was obtained laparoscopically, whereas previously investigators have evaluated whole ovaries so that it is possible that larger follicles were present but lay deeper at the cortical–medullary interface and therefore were not contained within these biopsies. Indeed, a recent study has shown that pre-antral follicles exist in and can be isolated from the ovarian medulla in young girls (Kristensen *et al*., 2011).

No difference was observed between the ability of follicles from frozen–thawed tissue and fresh tissue to activate and develop *in vitro*, although only two cryopreserved specimens were used and thus this study does not provide a robust analysis of tissue viability following cryopreservation in this young (≤16 years) age group. This is reassuring as all tissue collected for fertility preservation purposes will require to be cryopreserved and thawed prior to use. In this study cryopreserved tissue was slow frozen, and we are not aware of any data regarding the use of vitrification of ovarian tissue in this age group.

These results have implications for young children and adolescents who have had ovarian tissue collected for fertility preservation purposes prior to reaching sexual maturity. Currently, removal and cryopreservation of cortical tissue is the only option for pre-pubertal children facing loss of fertility through chemo/radiotherapy with later reimplantation of this tissue (Anderson and Wallace, 2011). In some patients tissue reimplantation is not desirable due to the risk of reintroducing malignant cells present in the stored tissue back into the cured patient (Dolmans *et al*., 2010; Rosendahl *et al*., 2010). This risk is clear in haematological malignancies, but can also occur with solid cancers (Abir *et al*., 2010). Development of a culture system which supports *in vitro* activation, growth and development of follicles in juvenile ovarian tissue used in conjunction with *in vitro* oocyte maturation and IVF techniques offers an alternative to reimplantation of potentially contaminated tissue collected from girls prior to sexual maturity. The present results suggest that methodologies suitable for fully mature adult ovarian tissue and isolated follicles are not appropriate for girls and adolescents and further refinement is required.

In summary, this study demonstrates the capacity of ovarian follicles in cortical biopsies from girls and adolescents to activate and grow to the secondary stage of development *in vitro*. Differences in the rate of activation and growth from adult follicles indicate intrinsic differences, which may in turn reflect compromised developmental competence. It is possible however that changes to the culture methodology may overcome this. In conjunction with *in vitro* maturation and IVF this is a potential method of preserving the fertility of girls who face sterilizing cancer treatments prior to sexual maturity. In addition, this work has identified a subgroup of non-growing follicles with abnormal oocyte morphology in pre-pubertal girls which are lost prior to the attainment of puberty.

## Authors' roles

R.A.A.: study design, data analysis, manuscript drafting and final approval. M.M.: study design, experimental procedures and data analysis, manuscript drafting and final approval. W.H.B.W.: study design, manuscript editing and final approval. D.F.A.: study design, experimental procedures and data analysis, manuscript editing and final approval. E.E.T.: study design, data analysis, manuscript drafting and final approval.

## Funding

Funded by MRC grants G0901839 and G1100357.

## Conflict of interest

None declared.
